# Repair of the Nonunion of the Lateral Process of the Talus Will Improve Osteoarthritis of the Subtalar Joint: A Case Report

**DOI:** 10.7759/cureus.84598

**Published:** 2025-05-22

**Authors:** Munekazu Kanemitsu, Tomoyuki Nakasa, Yasunari Ikuta, Akinori Nekomoto, Nobuo Adachi

**Affiliations:** 1 Department of Orthopaedic Surgery, Matsuyama Red Cross Hospital, Matsuyama, JPN; 2 Department of Artificial Joints and Biomaterials, Graduate School of Biomedical and Health Sciences, Hiroshima University, Hiroshima, JPN; 3 Department of Orthopaedic Surgery, Graduate School of Biomedical and Health Sciences, Hiroshima University, Hiroshima, JPN

**Keywords:** dismissed fracture, fracture of the lateral process of the talus, nonunion, subtalar osteoarthritis, surgical management

## Abstract

Fracture of the lateral process of the talus (FLPT) is a rare injury. FLPT is frequently missed or diagnosed late, often leading to nonunion, malunion, or subtalar osteoarthritis. We present a case of a 44-year-old male with schizophrenia who sustained an FLPT after falling downstairs. Initial radiographs missed the injury, and conservative treatment was pursued despite the presence of bone fragments. After four months, persistent pain led to further imaging, revealing subtalar joint osteoarthritis. Surgical intervention was required due to nonunion and large, displaced bone fragments. A 4 cm incision was made, and the fracture site was refreshed and fixed using screws with bone grafting. Postoperatively, the patient showed significant improvement, with complete bone union and enhanced joint space after one year. At the last follow-up (three years after surgery), the patient was able to work without difficulty. Late diagnosis and non-aggressive treatment can lead to poor prognosis, with late complications, including nonunion, malunion, bony overgrowth, and subtalar osteoarthritis. We presented a case of nonunion of the lateral process of the talus with osteoarthritis of the subtalar joint. A good clinical outcome was achieved by fixing the fracture and stabilizing the subtalar joint.

## Introduction

Fracture of the lateral process of the talus (FLPT) is a rare fracture [1]. FLPT accounts for 15% of snowboard-related ankle fractures and is also known as the "snowboarder's ankle" [2]. FLPT has often been neglected, with nearly half of these fractures reported to be diagnosed late and result in nonunion, malunion, or subtalar osteoarthritis [3-5]. We report the case of nonunion of FLPT with subtalar osteoarthritis that required surgical treatment.

FLPT is relatively rare and is often missed in diagnosis due to small bone fragments. However, even large bone fragments can be missed [5-8]. The lateral process of the talus is an important structure in stabilizing the subtalar joint [5], and misdiagnosing a large fragment can be a major hindrance. Although it can cause serious complications such as subtalar instability and osteoarthritis, there are few reports on the treatment of nonunion of FLPT [6-8]. We report a case in which the FLPT with a large bone fragment was missed due to worsening of schizophrenic symptoms and hospitalization for treatment, resulting in osteoarthritis of the subtalar joint, and achieved a good surgical outcome.

## Case presentation

**Figure 1:**
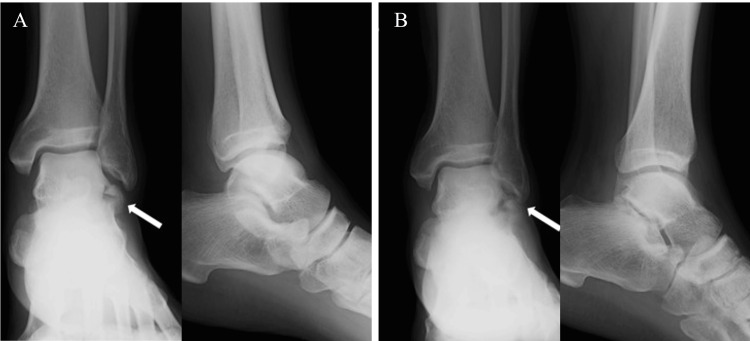
Plain radiograph findings. A: Plain radiographs at the time of injury. Fracture of the lateral process of the talus (FLPT) was observed. B: Plain radiographs at three months after the injury. Dislocation of bone fragments was increased.

**Figure 2:**
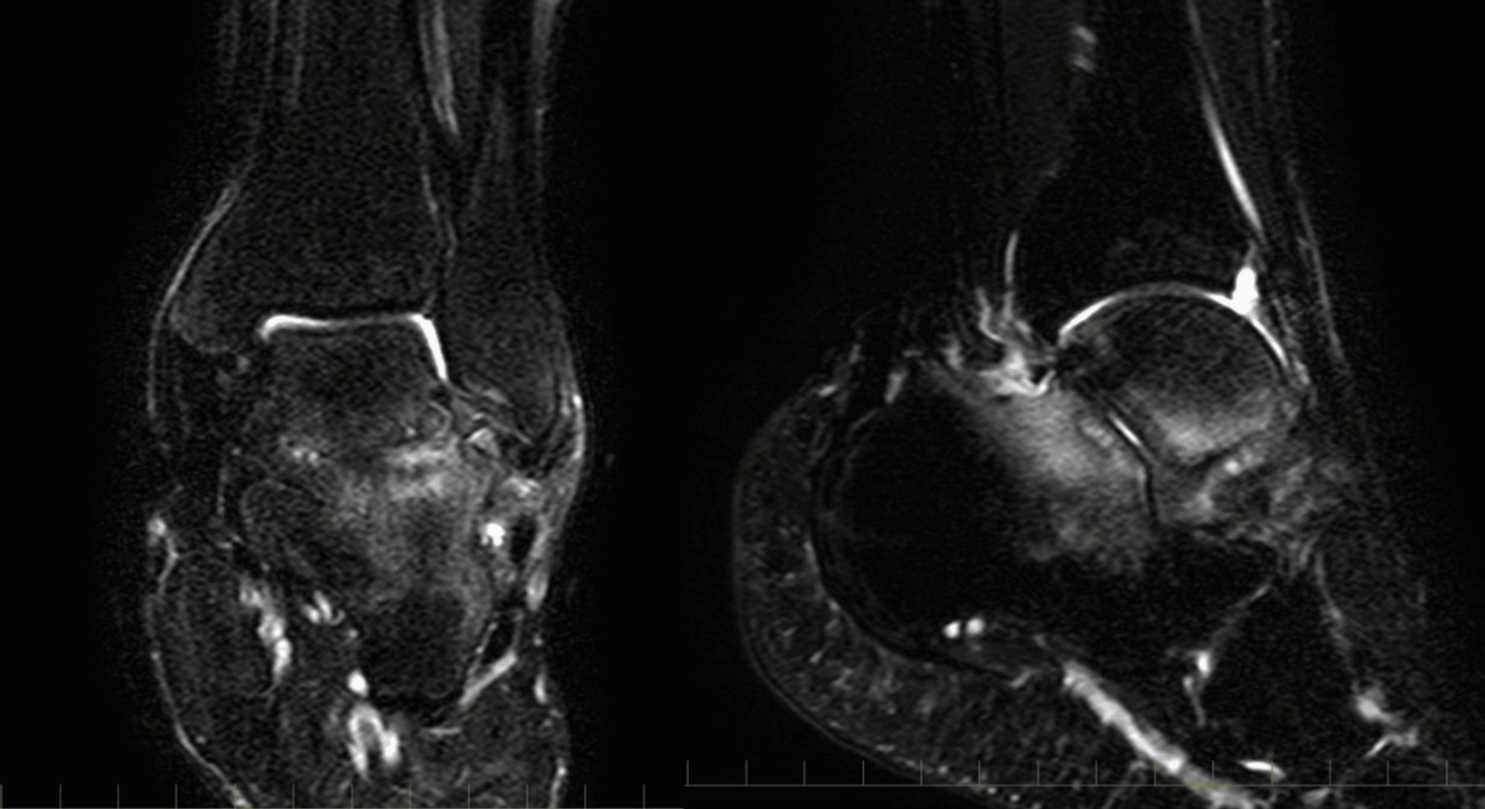
MRI finding. MRI shows a bone marrow lesion area at the subtalar joint.

**Figure 3:**
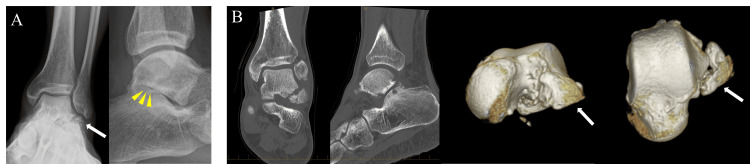
Plain radiograph and CT findings. A: Plain radiographs at the time of visit to our clinic. Nonunion of fracture of the lateral process of the talus (FLPT) and subtalar joint space narrowing were observed. B: CT showed a large bone fragment, subchondral bone sclerosis, and cystic lesion at the subtalar joint. C: 3D-CT showed a large fragment of fracture of the lateral process of the talus.

**Figure 4:**
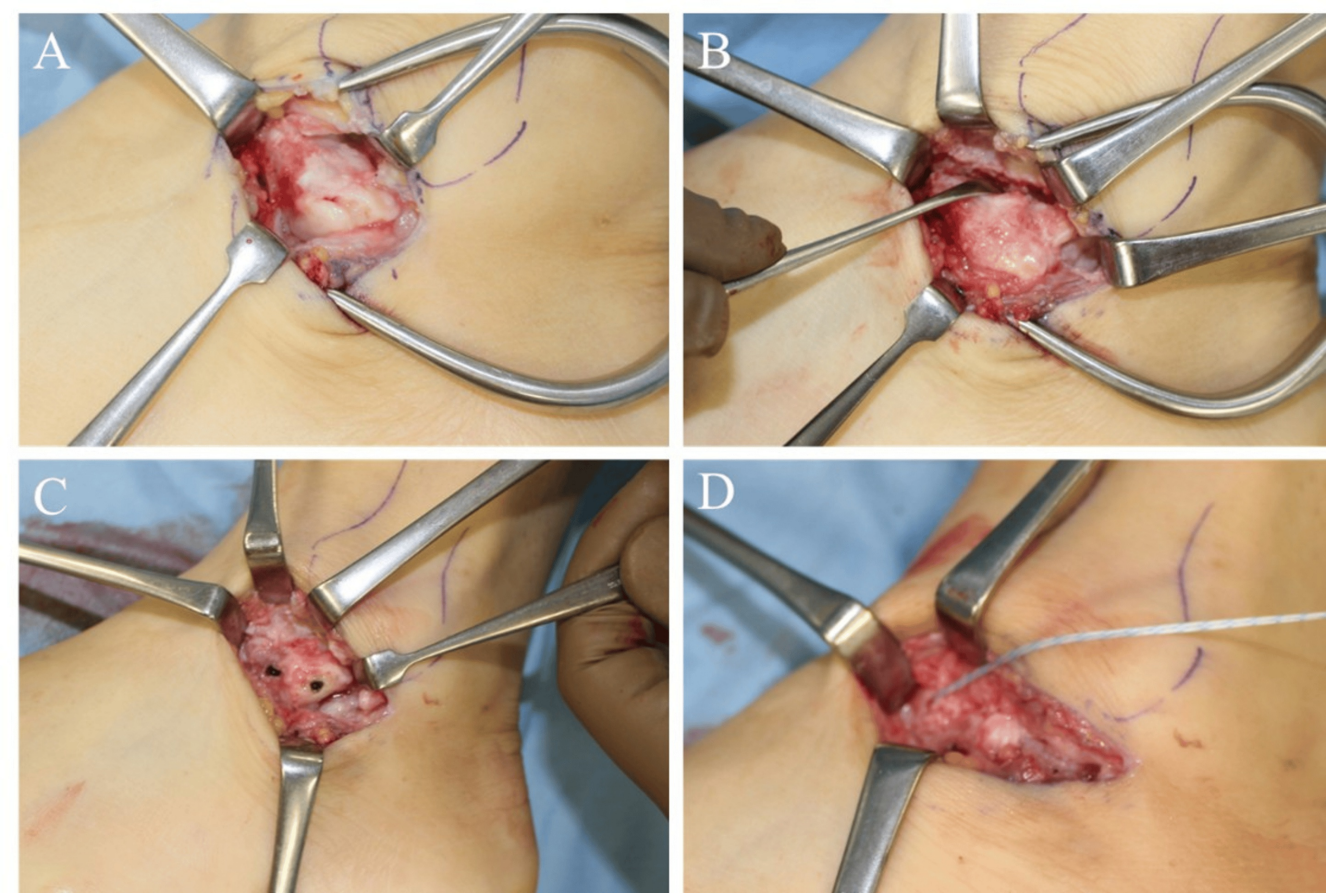
Intraoperative findings. A: Nonunion area was filled with scar tissue, and bone enlargement was observed. B: Scar tissue was resected. C: The bone fragment was fixed with headless screws. D: The anterior talofibular ligament was attached with a suture anchor.

The patient was a 44-year-old male with schizophrenia. He fell down the stairs and twisted his left ankle. He went to a nearby clinic. Plain ankle radiographs of anteroposterior images appeared to show bone fragments on the distal fibula (Figure 1A), and he underwent ankle fixation with a splint. One month after the injury, he was admitted to a psychiatric hospital for schizophrenia. Two months after the injury, ankle fixation was removed, and he underwent plain radiographs (Figure 1B) and computed tomography (CT), and dislocation of the bone fragment was observed, and he had ankle pain. However, he was treated conservatively because he had no local tenderness despite the fracture. Four months after the injury, his ankle pain had not improved. He underwent magnetic resonance imaging (MRI), which showed bone edema in the subtalar joint (Figure 2). Due to the appearance of pain in the subtalar joint that interfered with walking, he was referred to our hospital for surgical treatment four months after the injury. He complained of right ankle pain (VAS score was 8). The active range of motion of the ankle joint was 25 degrees in dorsal flexion and 45 degrees in plantar flexion. Before surgery, a CT scan was taken at our hospital, and the CT scan showed displaced large bony fragments at the lateral aspect of the talus and Kellgren-Lawrence grade 3 osteoarthritis of the subtalar joint (Figure 3). The surgical procedure was performed. A 4 cm skin incision was applied parallel to the talar axis. The anterior talofibular ligament (ATFL) was detached at the talar attachment as it interfered with fracture reduction. The nonunion area was filled with fibrotic scar tissue, and the scar tissue was removed (Figure 4 AB). The bone fragments were enlarged and required reshaping. In particular, the fracture surface side was shaved down to a smaller size, and good cancellous bone was observed. Cancellous bone harvested from the medial malleolus was grafted to the nonunion area and fixed with φ3.0 mm × 22 mm and φ3.0 mm × 28 mm headless screws (Dart-Fire, Stryker, Kalamazoo, MI, USA) (Figure 4C). Finally, a φ 1.8mm suture anchor (Q-fix, Smith & Nephew, Andover, MA, USA) was inserted into the ATFL attachment on the talar side, and the detached ATFL was secured to the talus (Figure 4D).

**Figure 5:**
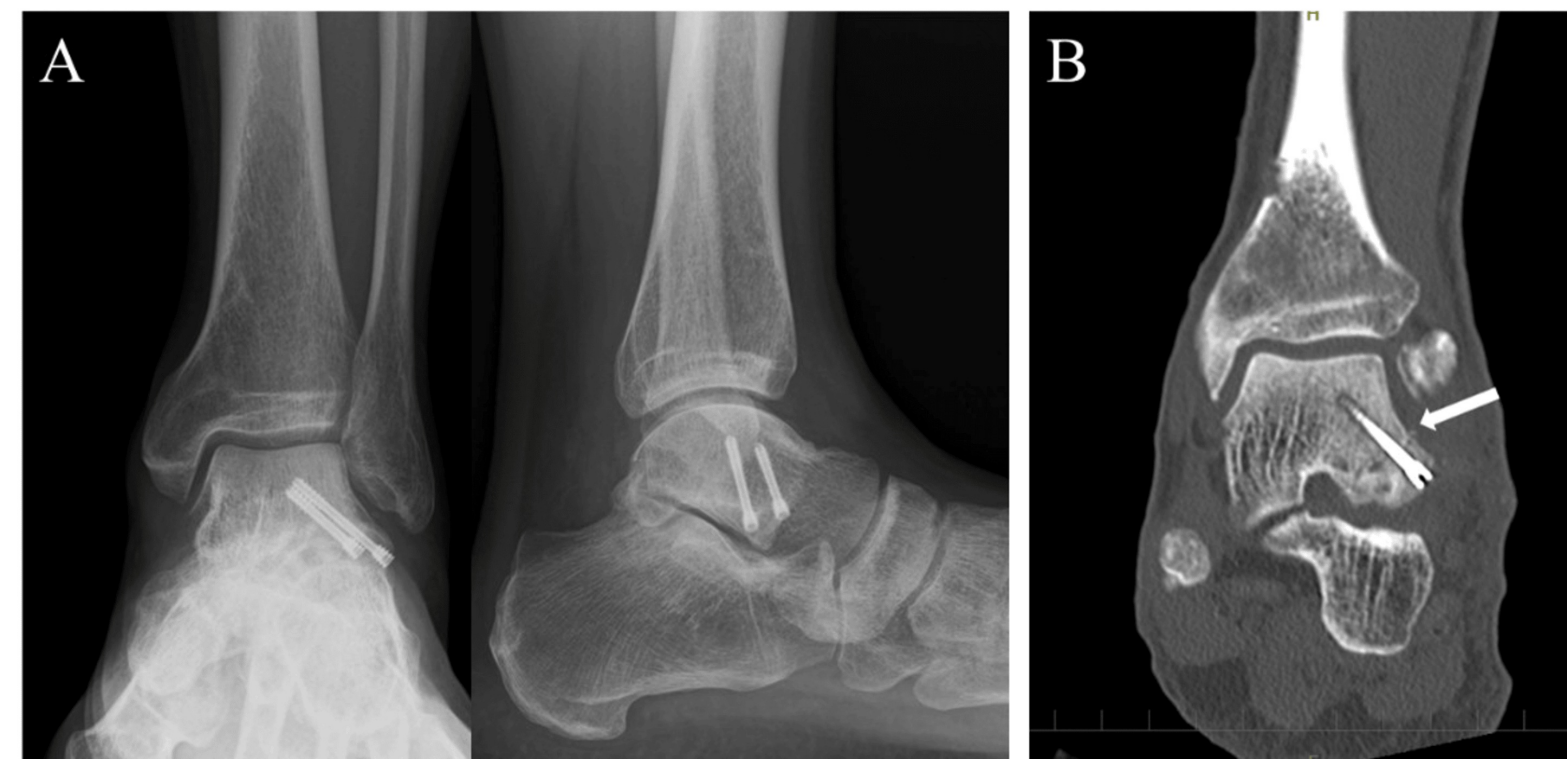
Plain radiographs and CT findings. A: Plain radiographs at one year after surgery. B: CT showed bone union.

**Figure 6:**
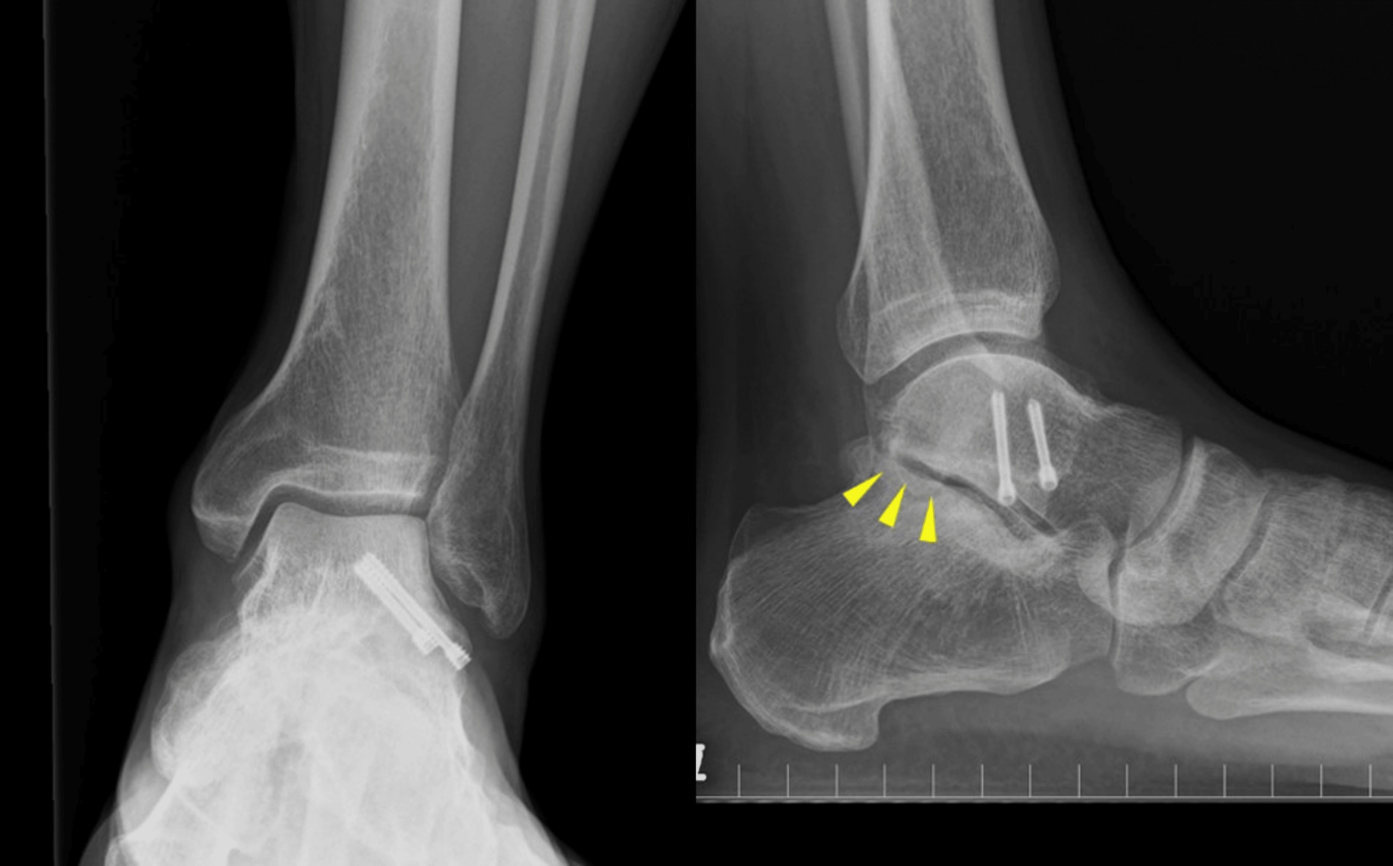
Plain radiograph findings. A: Plain radiographs at three years after surgery. Enlargement of subtalar joint space was observed.

Postoperative therapy consisted of cast immobilization and non-weight-bearing for four weeks, followed by range-of-motion training. Partial weight bearing was allowed at five weeks postoperatively, and full weight bearing at eight weeks postoperatively. At one year postoperatively, bone union was confirmed by CT, although cyst formation was observed on the fragment (Figure 5). At the last follow-up (three years after surgery), bone fusion of the nonunion area was observed on radiograph, and the subtalar joint space was enlarged (Figure 6). At the last follow-up (three years after surgery), the patient was able to work without difficulty in daily activities, although he had mild pain on landing (VAS was 1). The AOFAS Ankle-Hindfoot Scale was 90, and a self-administered foot evaluation questionnaire (SAFE-Q) was 93.3 in Pain and Pain-Related, 100.0 in Physical Functioning and Daily Living, 100.0 in Social Functioning, 100.0 in Shoe-Related, and 100.0 in General Health and Well-Being [9,10].

## Discussion

In this report, we experienced a case of non-union FLPT with subtalar osteoarthritis, and the subtalar joint enlargement was observed after surgery. Talar fractures account for approximately 0.1 to 0.85% of all fractures, and FLPT accounts for 20% of these fractures, making it very rare [1]. Late diagnosis and non-aggressive treatment will lead to a poor outcome, with severe complications, such as nonunion and malunion, leading to subtalar osteoarthritis [3,4].

The mechanism of injury in FLPT has not been elucidated, and several factors contribute to FLPT. Some reports have shown that FLPT is caused by forced dorsiflexion combined with foot inversion [3,11,12]. On the other hand, dorsiflexion of the foot combined with eversion or external rotation, axial impaction, eversion, and external rotation of the foot are also involved in FLPT [1,13,14]. Although there are many factors involved in the development of FLTP, and the fracture site, fragment size, degree of comminution, and displacement vary with the type and intensity of the injury, it is more important to avoid missed diagnoses and to provide timely and accurate diagnosis and treatment than to match the mechanism of injury to the characteristics of the fracture. [5]. Although the details of this case are unknown, it is likely due to dorsiflexion of the ankle and axial pressure applied to the ankle in inversion or eversion due to a fall downstairs.

Hawkins classified FLPT into three groups: type 1, simple fracture involving both the talofibular and posterior subtalar articular surfaces; type 2, comminuted fracture; and type 3, the so-called chip fracture, in which no articular surface is injured [11]. Our patient had a displaced Hawkins type I fracture. Surgery is performed by resection for small bone fragments and open reduction and internal fixation with screws or Kirschner wire for large bone fragments, and good results have been reported [5]. However, FLPT has often been neglected, resulting in nonunion, malunion, bony overgrowth, and subtalar osteoarthritis [3]. Nearly half of FLPT cases are reported to be diagnosed late [4].

Although radiography is currently the preferred examination, small fragments may overlap with other tissues or structures and be difficult to detect on plain radiographs, resulting in a missed diagnosis [5]. Nonunion is also associated with a poor outcome in 54% of cases and with large intra-articular fragments in 70% of cases [15]. Nonunion fractures cause ankle and subtalar instability, leading to impingement of the talocalcaneal and talofibular articulations [7,8]. This case was a nonunion case with large intra-articular fragments, which would likely have resulted in a poor outcome. There are few reports on the treatment of nonunion of FLPT. Mui et al. reported a case of postoperative anterior facet syndrome and revision surgery after bone grafting and open reduction and internal fixation for nonunion of FLPT [6]. Wang et al. reported a case of lateral impingement syndrome after nonunion of an FLPT and performed a subtalar fusion and Chevron procedure [7]. Killen et al. reported two cases of patients with revision surgery due to nonunion after conservative treatment [8]. Good postoperative results were reported in these cases. In this case, FLPT caused subtalar joint instability, which resulted in subtalar joint osteoarthritis. Stabilization of the subtalar joint eliminated the abnormal loading and promoted repair by fibrocartilage. A good surgical result was achieved by fixing the fracture and stabilizing the subtalar joint without any complications, such as lateral impingement syndrome.

This report has some limitations. First, this is a short-term follow-up case, and further follow-up is considered necessary to check the progression of subtalar osteoarthritis. Second, since this is a single case, it has not been possible to compare the results with those of other surgical techniques or treatment methods. Further study of more cases is needed.

## Conclusions

FLPT is often dismissed, and its nonunion leads to late complications such as subtalar osteoarthritis. Therefore, it is important to know that an ankle sprain can cause FLPT and to diagnose it properly.
